# Global trends in research of nasopharyngeal carcinoma: a bibliometric and visualization analysis

**DOI:** 10.3389/fonc.2024.1392245

**Published:** 2024-07-02

**Authors:** Guilin An, Jie Liu, Ting Lin, Lan He, Yingchun He

**Affiliations:** ^1^ Graduate School, Hunan University of Chinese Medicine, Changsha, Hunan, China; ^2^ Hunan Provincial Engineering and Technological Research Center for Prevention and Treatment of Ophthalmology and Otolaryngology Diseases with Chinese Medicine and Protecting Visual Function, Hunan University of Chinese Medicine, Changsha, Hunan, China; ^3^ The First Clinical College of Traditional Chinese Medicine, Hunan University of Chinese Medicine, Changsha, Hunan, China; ^4^ Hunan Provincial Key Laboratory for the Prevention and Treatment of Ophthalmology and Otolaryngology Diseases with Traditional Chinese Medicine, Hunan University of Chinese Medicine, Changsha, Hunan, China

**Keywords:** nasopharyngeal carcinoma, bibliometric analysis, research trends, VOSviewer, CiteSpace

## Abstract

**Objective:**

This study aims to assess the current research status, focus areas, and developmental trends in nasopharyngeal carcinoma (NPC) through a bibliometric analysis.

**Methods:**

Articles focusing on NPC published from 2000 to 2023 were retrieved from the Web of Science database. VOSviewer and CiteSpace were used for bibliometric and visual analysis.

**Results:**

A total of 14516 related publications were retrieved. There has been a steady increase in the number of NPC-related publications from 2000 to 2023. China was the dominant country in this field with 8948 papers (61.64%), followed by the USA (2234, 15.39%). Sun Yat-sen University was the most influential institution, while Ma J was the most prolific author. Furthermore, *Head And Neck-journal For The Sciences And Specialties Of The Head And Neck* was the most prolific journal. *International Journal of Radiation Oncology Biology Physics* had the highest total citation counts. "Introduction chemotherapy", "Concurrent chemotherapy", "Epithelial-mesenchymal transition", "Cancer stem cells", "MicroRNAs", "LncRNA", "Exosomes", and "Biomarker" were the most common keywords. The reference "Chen YP, 2019, Lancet" had the highest citations and strong outbreak value.

**Conclusion:**

The past two decades have witnessed a significant increase in research on NPC. The optimization of treatment mode is the most widely studied aspect at present. The mechanism of occurrence and development and the most favorable diagnostic and therapeutic targets are the research hotspots in the future.

## 1 Introduction

Nasopharyngeal carcinoma (NPC) is a malignant tumor originating from the mucosal epithelium of the nasopharynx that exhibits uniqueness in epidemiology, histology, and virology. NPC has an obvious geographical distribution, and more than 77% of the incidence rate of NPC occurs in East Asia and Southeast Asia, especially in southern China (1). Epstein-Barr virus (EBV) is generally considered a significant cause of NPC, and lifestyle habits such as eating preserved food, smoking, and drinking alcohol are associated with an increasing risk of NPC (2). Nonetheless, the exact pathogenesis of NPC still needs to be elucidated. NPC is mostly a poorly differentiated squamous cell carcinoma that occurs in the fossa of Rosenmüller (3). Compared to the patients with late-stage NPC, the overall 5-year survival rate of patients with early-stage exceeds 90% (4). However, due to the hidden location of NPC, most patients have already reached extremely poor staging when diagnosed. Even if patients receive active treatment, lesions in the local nasopharyngeal and cervical lymph nodes of cancer are still more likely to remain or recur, which greatly affects the overall survival (OS) rate of patients (5). Therefore, NPC causes serious health burden.

NPC is a topic of continuous concern. Many studies related to etiology, histopathology, epidemiology, and treatment are published annually. However, the number of studies has grown rapidly, and cannot keep up with the latest findings on all issues. Bibliometric analysis is an emerging tool that quickly explores the structure and trends of a field through specific computational methods and visual analysis. In recent years, this method has been widely applied in the analysis of massive scientific research data (6). Bibliometric analysis of medical paper data is necessary and valuable, as it can objectively reflect the current development status, research hotspots, and future trends (7). Although there have been bibliometric studies on NPC before, we cannot identify research hotspots due to temporal updates and methodological variations (8–10). Therefore, this study evaluates the global publications in the field of NPC from 2000 to 2023 in order to provide new insights into the research trends of NPC. Specifically, this study mainly used VOSviewer and CiteSpace software to capture the growth trend of NPC publications, measure the contributions of countries, institutions, journals and authors, and determine the main keywords and important literatures, so as to reveal the research hotspots and development trends in this field.

## 2 Methods

### 2.1 Data acquisition

Web of Science is the primary research platform for hard science, social sciences, arts, and humanities information, as well as the independent global citation database of the world’s most trusted publishers (11). To improve data representativeness and accessibility, we retrieved the Web of Science core collection (WoSCC).

**Figure 1:**
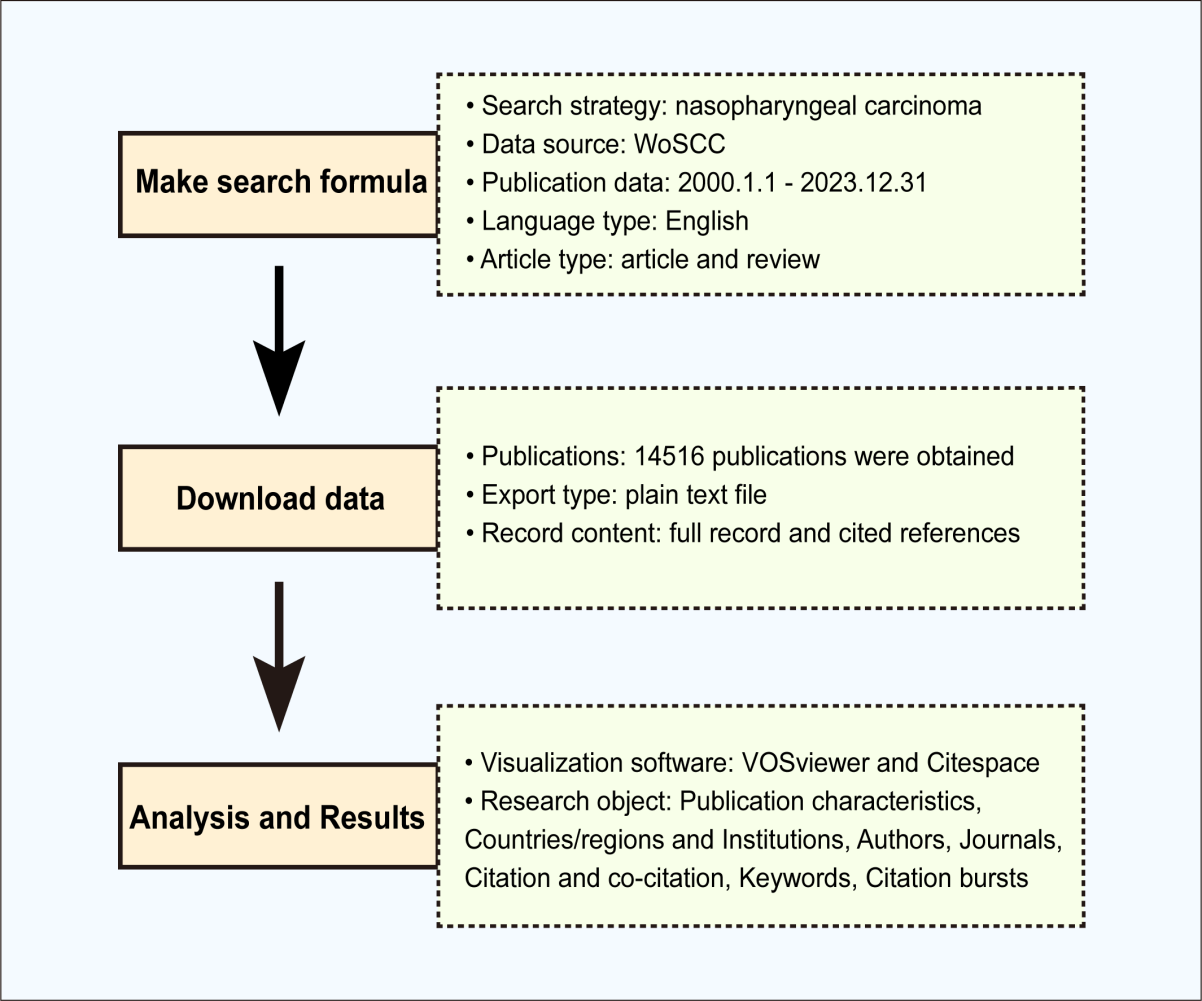
Research process applied in this study.

The following search terms were employed to retrieve literature from WoSCC: TS = (“nasopharyngeal carcinoma”). The publication date was from 2000-01-01 to 2023-12-31. The language type is selected as English, and the article type is selected as article and review. The search results were exported with a “Plain Text file” and the record content was chosen “Full Record and Cited References” and stored in “download_*.txt” format. Two researchers independently completed literature screening, data extraction, and analysis to ensure the reliability of the results. The retrieval framework is shown in **Figure 1**.

### 2.2 Bibliometrics and visualization analysis

Bibliometric analysis was conducted using four tools, namely VOSviewer (version 1.6.19), CiteSpace (version 6.2. R6), R (version 4.2.2), and GraphPad Prism (version 9.5.1).

VOSviewer is a distance-based bibliometric tool that has advantages in visualizing bibliometric networks (12). VOSviewer was used to analyze the countries, institutions, and authors with the most production/collaboration, as well as the journals with the most citations and the most frequently appearing keywords, and generate a visual knowledge graph. Link strength (LS) represents the strength of cooperation between nodes, while total link strength (TLS) reflects the overall level of cooperation.

CiteSpace is a Java application developed by Professor Chen Chaomei for bibliometric analysis, which focuses on the dynamic visualization of bibliometrics and reflects the evolution of bibliometric networks over time (13, 14). In this study, CiteSpace was used to identify the highly cited keywords and references with the strongest citation burst over a certain period.

Bibliometrix is an R package that includes the functionality of scientific quantitative research (15). This study used it to summarize the publication and citation count of bibliometric analysis and visualize a Three-Fields Plot.

GraphPad Prism was used to generate a line graph of the number of publications, cumulative number of publications, citation count, and H-index.

## 3 Results

### 3.1 Publication characteristics

**Figure 2:**
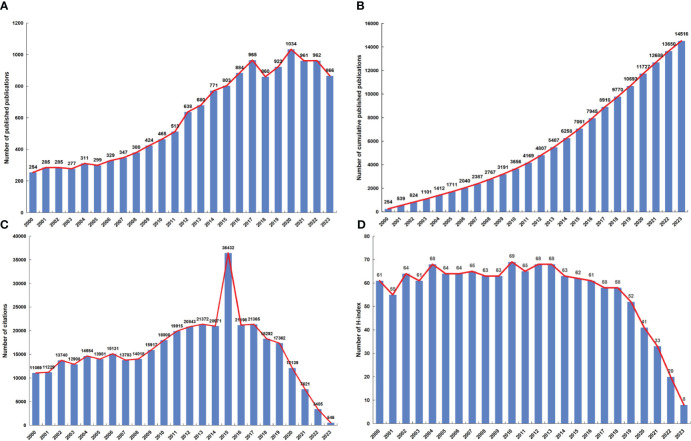
**(A)** The global annual number of publications related to NPC research. **(B)** The global annual number of cumulative publications related to NPC research. **(C)** The global annual number of citations of the publications related to NPC research. **(D)** The global annual H-index values of the publications related to NPC research.

A total of 14516 publications related to NPC were obtained from the WoSCC. The distribution of annual publication volume from 2000 to 2023 is shown in **Figure 2A**. The number of annual publications showed an overall increasing trend, indicating that attention to the field of NPC increased. The number of published articles peaked in 2020 (1034, 7.12%). The cumulative number of publications increased steadily from 2000 to 2023 (**Figure 2B**). From 2009 to 2019, citations were relatively high (**Figure 2C**). The annual H-index from 2000 to 2018 was above 55, while the H-index from 2019 to 2023 declined (**Figure 2D**).

### 3.2 Countries/regions and institutions

**Figure 3:**
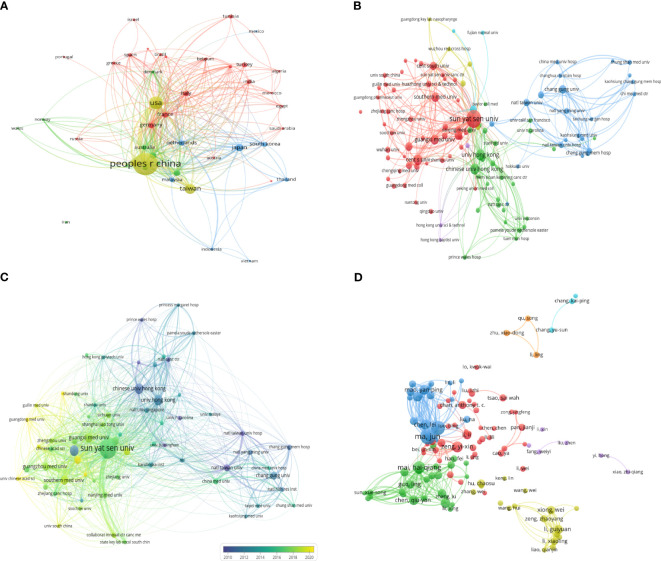
The coauthorship network map of countries/regions **(A)**, and institutions **(B)**. **(C)** The coauthorship overlay map of institutions. **(D)** The coauthorship network map of authors.

113 Counties/regions have contributed to research related to NPC. **Figure 3A** shows 44 countries/regions with more than 15 submissions and 588 collaboration instances. China had the strongest international cooperation network (TLS=1890) and the closest cooperation with the United States (LS=808).

Next, we analyzed the number of publications and total citations for the 10 most productive countries/regions. As shown in **Table 1**, China had the most publications (8948, 61.64%), followed by the USA (2234, 15.39%) and the Taiwan(1191, 8.20%). Each of them published fewer than 1000 articles. In addition, China had the highest number of citations.

**Table 1:** The top 10 most productive countries/regions regarding NPC from 2000 to 2023.

Rank	Country/region	Publication counts	Publications weight	Total citations
1	China	8948	61.64%	198944
2	USA	2234	15.39%	111964
3	Taiwan	1191	8.20%	34602
4	Japan	543	3.74%	15724
5	Singapore	415	2.86%	15616
6	England	360	2.48%	17249
7	France	339	2.34%	28983
8	Germany	332	2.29%	10151
9	Canada	295	2.03%	13544
10	Italy	257	1.77%	8382

A total of 7478 institutions contributed to NPC research. **Figure 3B** shows 109 institutions with more than 50 documents and 1928 instances. Sun Yat-sen University had the largest cooperative network (TLS=2036). The top 10 most productive institutions are shown in **Table 2**. Sun Yat-sen University was the most productive institution (2116, 14.58%), followed by Central South University (883, 6.08%), and the University of Hong Kong (600, 4.13%). Sun Yat-sen University also had the highest number of total citations (58162).

**Table 2:** The top 10 most productive institutions regarding NPC research from 2000 to 2023.

Rank	Institution	Publication counts	Publications weight	Total citations
1	Sun Yat-sen University	2116	14.58%	58162
2	Central South University	883	6.08%	20319
3	The University of Hong Kong	600	4.13%	25167
4	The Chinese University of Hong Kong	584	4.02%	30898
5	Guangxi Medical University	449	3.09%	7282
6	Southern Medical University	430	2.96%	9087
7	Fudan University	385	2.65%	9783
8	Guangzhou Medical University	338	2.33%	8305
9	Chang Gung University	331	2.28%	9385
10	Fujian Medical University	286	1.97%	5326

In the overlay network analyzed by the co-authors, the size of the circle represents the number of publications, and the color represents the average start year of each institution’s publications in a particular field of study. As shown in **Figure 3C**, the study identified 66 institutions with at least 75 publications. The Prince of Wales Hospital, the University of Hong Kong, and the Chinese University of Hong Kong started earlier. In contrast, Sun Yat-sen University, Guangzhou Medical University, and Southern Medical University recently conducted research in this area.

### 3.3 Authors

**Figure 4:**
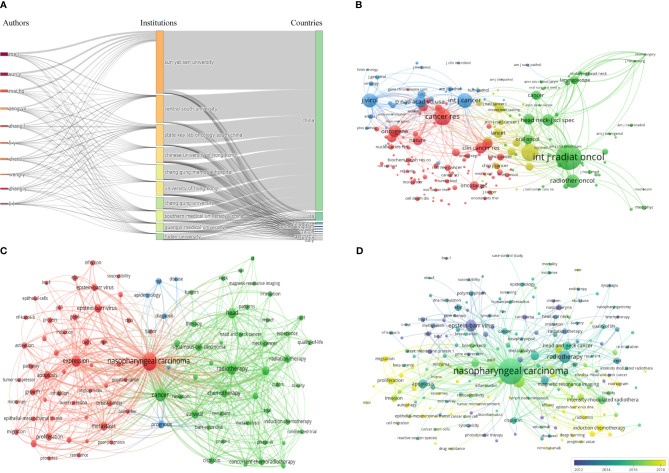
**(A)** Three Fields Plot. **(B)** The co-citation network map of journals. **(C)** Keyword co-occurrence network diagram of NPC. **(D)** Keyword co-occurrence average years network diagram of NPC.

A total of 53787 authors contributed to publications related to NPC. **Table 3** shows the top 10 authors ranked by the number of published articles according to co-author analysis. Professor Ma J had the highest efficiency (305, 2.10%), followed by Sun Y (259, 1.78%) and Mai HQ (191, 1.32%), all from Sun Yat-sen University. In addition, Ma J was the author with the highest total citations and H-index. **Figure 3D** shows a collaborative map among 118 researchers, with a minimum of 40 papers. Ma J had the highest number of collaborative relationships with other authors (TLS=1869). **Figure 4A** visually illustrates the inflow and outflow of ten authors, affiliated institutions, and countries who have contributed to NPC research from 2000 to 2023.

**Table 3:** The top 10 most productive authors regarding NPC research from 2000 to 2023.

Rank	Authors	Publication counts	Publications weight	Total citations	H-Index
1	Ma J	305	2.10%	11714	67
2	Sun Y	259	1.78%	9616	57
3	Mai HQ	191	1.32%	4901	45
4	Zeng YX	160	1.10%	8095	59
5	Mao YP	135	0.93%	5301	43
6	Chen QY	133	0.92%	2979	35
7	Tang LL	133	0.92%	5058	42
8	Chen L	130	0.90%	4980	42
9	Guo X	130	0.90%	3306	39
10	Li GY	129	0.89%	5277	49

### 3.4 Journals

Publications on NPC research were published in 1709 journals. **Table 4** shows the top 10 most widely published journals and their latest issues of JournaI Citation Reports (JCR), Impact Factor (IF), and H-index. *Head And Neck-journal For The Sciences And Specialties Of The Head And Neck* had the highest number of articles (publication counts=373), followed by *International Journal of Radiation Oncology Biology Physics* (publication counts=340) and *PLoS One* (publication counts=292). *International Journal of Radiation Oncology Biology Physics* had the highest total citation counts (23825) and average citation counts (70.07). In terms of JCR, most journals were classified as Q1 and Q2 (80%). In terms of journal direction, these 10 journals cover head and neck diseases, oncology, tumor radiotherapy, etc. IF is an important parameter for evaluating the value of journals. *International Journal of Radiation Oncology Biology Physics* had the highest IF (7), followed by *International Journal of Cancer* (6.4) and *Radiotherapy and Oncology* (5.7). In addition, *PLoS One* had the highest H-index (268), followed by *International Journal of Radiation Oncology Biology Physics* (228) and *International Journal of Cancer* (212). The journal co-citation network map is shown in **Figure 4B**. The top 3 co-cited journals were *International Journal Of Radiation Oncology Biology Physics* (26354 citations), *Journal Of Clinical Oncology* (13104 citations), and *Cancer Research* (12992 citations).

**Table 4:** The top 10 most productive journals regarding NPC research from 2000 to 2023.

Rank	Journal title	Publication counts	Total citations	Average citation	JCR (2023)	IF (2023)	H-Index
1	Head And Neck-journal For The Sciences And Specialties Of The Head And Neck	373	7914	21.22	Q1	2.9	113
2	International Journal of Radiation Oncology Biology Physics	340	23825	70.07	Q1	7	228
3	PLoS One	292	8197	28.07	Q2	3.7	268
4	Oncotarget	280	7710	27.54	\	\	91
5	Oral Oncology	263	5524	21.00	Q1	4.8	101
6	Frontiers in Oncology	243	1541	6.34	Q2	4.7	60
7	Radiotherapy and Oncology	236	8792	37.25	Q1	5.7	140
8	BMC Cancer	202	4562	22.58	Q2	3.8	111
9	International Journal of Cancer	180	7602	42.23	Q1	6.4	212
10	Oncology Letters	176	1953	11.10	Q3	2.9	38

### 3.5 Citation and co-citation analysis

**Figure 5:**
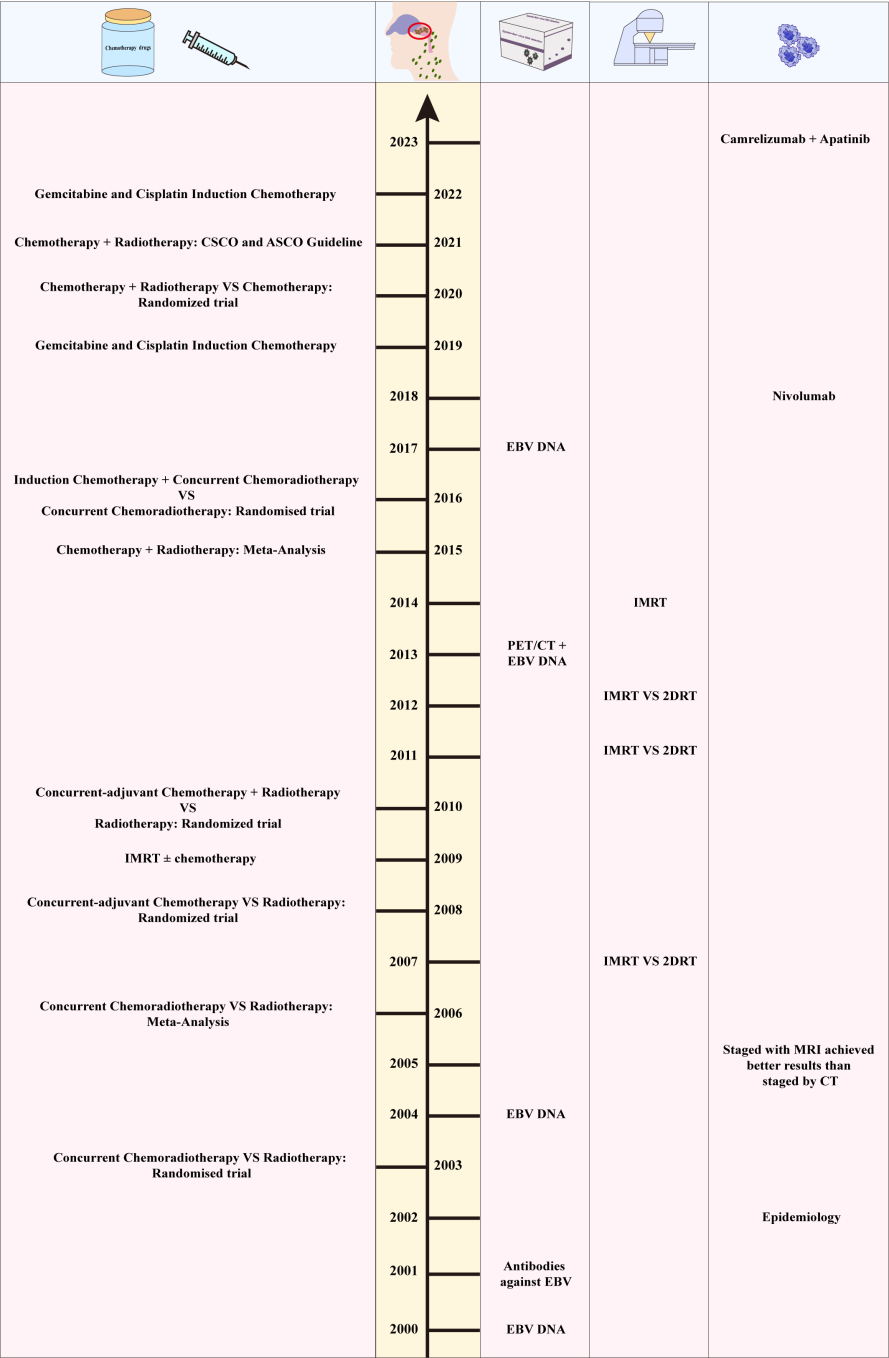
The timeline of NPC research.

Citation analysis is a valuable way to evaluate the most cited articles, and the number of citations can reflect the impact of an article in a particular field of research. **Figure 5** and **Supplementary Table 1** list the most cited publications on NPC each year from 2000 to 2023. Among the 24 articles, 8 were published in *Journal of Clinical Oncology*, 4 in *the New England Journal of Medicine*, 4 in *International Journal of Radiation Oncology Biology Physics*, 2 in *Radiation and Oncology*, and 2 in *Lancet Oncology*. Others were published in *JAMA Oncology*, *Cancer Research*, *Jnci-journal of the National Cancer Institute*, and *Seminars in Cancer Biology*. Furthermore, 24 articles included 11 studies on chemotherapy, 5 on EBV, 4 on intensity-modulated radiotherapy (IMRT), 2 on immunotherapy, 1 on epidemiology, and 1 on retrospective analysis.

This study also analyzes the most frequently cited references. **Supplementary Table 2** lists the 10 most cited articles, all of which were cited more than 400 times. Specifically, the article titled “Nasopharyngeal Carcinoma” published in *Lancet* in 2019 had the highest citation count (1092). Other articles included 2 reviews on NPC, 2 reviews on NPC epidemiology, 3 phase III randomized intergroup studies on chemotherapy in different treatment stages of patients with NPC, 1 clinical study on IMRT, and 1 global cancer data analysis.

### 3.6 Keywords

This study counted 29447 keywords, and **Figure 4C** shows the network visualization of some keywords (with a minimum occurrence of 50, including a total of 86 keywords). The top 10 most frequently used keywords are “nasopharyngeal cancer”, “cancer”, “radiation”, “expression”, “head”, “survival”, “intensive modulated radiation”, “chemotherapy”, “metastasis”, and “Epstein-Barr virus”.

**Figure 4D** shows the overlay visualization of author keywords. The term marked in purple indicates an average publication year of 2012 or earlier, while the term marked in bright yellow appears after 2018. “Epstein-Barr virus”, “p53”, and “late member protein 1” were the main topics in the early stages. The keywords “introduction chemotherapy”, “concurrent chemotherapy”, “epithelial-mesenchymal transition”, “cancer stem cells”, “lncRNA”, “microRNAs”, “exosomes”, and “biomarker” appeared relatively late during the research period.

**Figure 6:**
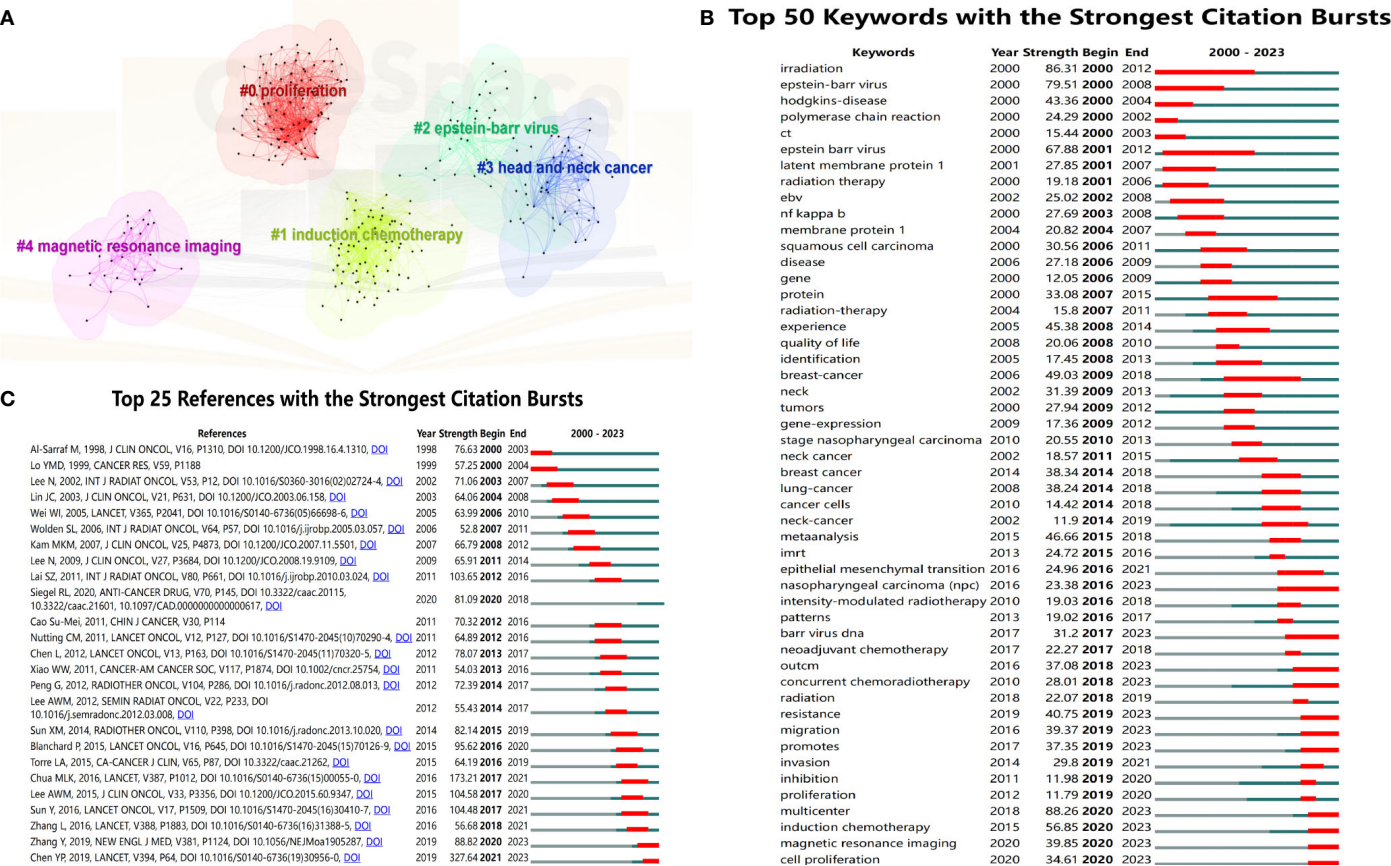
**(A)** Five representative clusters of keyword clustering from 2019 to 2023. The top 50 keywords **(B)** and 25 references **(C)** with the strongest citation bursts.

To obtain the research hotspots in the field of NPC in recent years, this study conducted cluster analysis on keywords within the past 5 years (2019-2023) and finally formed 5 representative clusters (**Figure 6A**). The clustering map shows that the modularity value is 0.475 (> 0.3), indicating a significant clustering structure; the silhouette value is 0.8049 (> 0.5), indicating a reasonable clustering. Among the 5 cluster labels, the largest cluster size is #0 (Promotion, Size=100), followed by #1 (Introduction chemotherapy, Size=78), #2 (Epstein-Barr virus, Size=43), #3 (Head and neck cancer, Size=40), and #4 (Magnetic resonance imaging, Size=30). Based on clustering vocabulary analysis, the research focus of NPC mainly includes molecular mechanisms, treatment strategies, EBV, and diagnostic methods.

### 3.7 Citation bursts

**Figure 6B** shows the top 50 keywords with the strongest citation bursts, and the red line represents the duration of the burst. The keywords “radiation” (2000-2012) and “Epstein-Barr virus” (2001-2012) received considerable attention in the past period. The vocabulary of “concurrent chemotherapy” (2018-2023), “resistance” (2019-2023), “migration” (2019-2023), “promotes” (2019-2023), “multicenter” (2020-2023), “introduction chemotherapy” (2020-2023), “magnetic response imaging” (2020-2023), and “cell promotion” (2020-2023) received high attention in recent years. They may still be research hotspots in the future.

**Figure 6C** shows the top 25 references with the highest citation burst. The article “Nasopharyngeal Carcinoma” published by Chen YP et al. in *Lancet* had a strong outbreak value and is still ongoing. Meanwhile, the citation explosion of “Gemcitabine and Cisplatin Induction Chemotherapy in Nasopharyngeal Carcinoma” published by Zhang Y et al. in *the New England Journal of Medicine* is also ongoing. This article and Sun Ying et al.’s article are both prospective randomized controlled studies targeting locally advanced NPC. The results showed that the use of a GP regimen (gemcitabine plus cisplatin) and an improved TPF regimen (docetaxel, cisplatin, 5-FU) for induction chemotherapy significantly improved OS of patients.

## 4 Discussion

### 4.1 Trends in the development of NPC-related research

To our knowledge, this is the first study to conduct a comprehensive bibliometric analysis of publications related to NPC from 2000 to 2023. Our research findings indicate that the number of annual publications in this field is on the rise. The highest citation count was in 2015, and the highest H-index was in 2010. The decrease in citation frequency and H-index after 2018 may be attributed to the approaching data collection time.

### 4.2 Contributions by countries, institutions, journals, and authors

In this bibliometric analysis, we analyzed the most influential countries, institutions, authors, and journals in the field of NPC. China was the major contributor to this research field, with the highest number of publications, citations, and international cooperation. The top 10 institutions and 10 authors with the highest productivity are all from China. Sun Yat-sen University was the most influential institution, while Ma J was the most prolific author. NPC is common in some ethnic groups, especially in southern China, Hong Kong, and Southeast Asia (16). Therefore, as a high-risk area for NPC, China ranks first in the world in terms of scholars and institutions conducting research in this field. The above results are consistent with the results of two previous studies (8, 10). It is worth noting that in another study with a large statistical time span (1970-2018), the most influential institutions and authors were the Prince of Wales Hospital and Chan ATC (9). But our research results show that the Prince of Wales Hospital and Chan ATC are the early founders of NPC (**Figure 3C**; **Supplementary Figure 1**). In addition, our results show that *Head And Neck Journal For The Sciences And Specialties Of The Head And Neck*, *International Journal of Radiation Oncology Biology Physics*, and *PLoS One* were considered the most influential journals in this field. *Head And Neck Journal For The Sciences And Specialties Of The Head And Neck* had the highest number of publications; *International Journal of Radiation Oncology Biology Physics* had the highest citation counts and journal IF; *PLoS One* had the highest H-index. Although these results are different from previous research results, in general, *International Journal of Radiation Oncology Biology Physics* has always been an influential journal in the NPC field (8–10). Because NPC is sensitive to radiotherapy, relevant professional journal continues to attract researchers’ attention.

### 4.3 Research hotspots and frontiers

Citation, co-citation, and keyword analysis offer insights into the hotspots and developmental trends in NPC-related research. Compared with the previous NPC bibliometric research findings, this study proposes some new conclusions (8–10). Firstly, the citation and co-citation results show that the development of effective treatment schemes (radiotherapy, concurrent chemoradiotherapy, induction chemotherapy, and adjuvant chemotherapy) has always been a research hotspot, which is similar to the previous research results. But currently, researchers are working to reduce the area of radiotherapy and identify the most suitable candidates for chemotherapy, aiming to make treatment plans more targeted. At the same time, targeted therapy and immunotherapy are increasingly gaining attention from researchers. Secondly, the keyword analysis results show that in recent years, researchers have focused on “epithelial-mesenchymal transition”, “cancer stem cells”, “lncRNA”, “microRNAs”, and “exosomes”, aiming to identify key diagnostic and therapeutic biomarkers for NPC. But in previous studies, researchers have paid more attention to EBV, gene expression, RNA guided endonuclease. Thirdly, keyword burst analysis and citation burst analysis mentioned “EBV” and “MRI”, indicating that they are of great significance for the diagnosis and therapeutic evaluation of NPC. Fourthly, combined with the latest literature, we find that the global concept of NPC has changed, but previous studies have not mentioned this problem.

#### 4.3.1 Clinical treatment strategy

Due to NPC cells being sensitive to radiotherapy, 2-dimensional radiotherapy (2DRT) was used earlier to treat NPC. However, the anticancer efficiency of 2DRT is limited and the side effects are obvious, resulting in many patients refusing treatment. IMRT is an advanced external radiotherapy technology, which can precisely adjust the radiation dose distribution so that the tumor receives a higher dose and the normal tissue receives a lower dose. Lee et al. showed that IMRT provides excellent tumor target coverage and allows for high-dose delivery to the target while significantly preserving salivary glands and other key normal tissues nearby (17). Kam, M. K et al. showed that IMRT significantly reduces the incidence of severe late-onset dry mouth (18). Lai SZ et al. conducted a retrospective analysis of data from 1276 patients with non-metastatic NPC confirmed by biopsy, and the results showed that IMRT has better local tumor control rates than traditional 2DRT, especially in patients with early T-stage (19). Peng, G et al. found that IMRT improves local recurrence-free survival, especially in patients with advanced NPC (20). Research by Sun, X et al. showed that the survival outcomes of IMRT treatment for NPC are excellent, but distant metastasis is a concern (21). However, the potential harm of IMRT to oral muscles, thyroid, and cervical tissues cannot be overlooked. Recently, Huang, C. L et al. reported that Upper-Neck Irradiation (UNI), a novel radiotherapy approach tailored for patients with stage N0-1 NPC, significantly protects cervical organs and tissues while maintaining therapeutic efficacy, in contrast to Whole-Neck Irradiation (WNI) (22). Mao, Y. P et al. found that for newly diagnosed adult patients (18-65 years old) with non-keratinizing, non-distant metastatic, non-medial retropharyngeal lymph node (MRLN) metastasis, preserving the MRLN reduces the incidence of dysphagia without altering outcomes when compared to standard radiotherapy (23). Consequently, the precision of radiotherapy for NPC is continuously improving.

Chemotherapy is also an important method for treating cancer, and the 0099 study laid the foundation for the combination of radiotherapy and chemotherapy in the treatment of NPC. Al-Sarraf M et al.’s research indicated that patients in the combination group of radiotherapy and chemotherapy have a higher OS rate and better safety compared to those in the simple radiotherapy group (24). Another study found that although IMRT has good therapeutic effects on NPC with or without chemotherapy, patients with poor TNM staging still have a higher rate of distant metastasis (25). Lin JC et al. showed that concurrent chemoradiotherapy (CCRT) is superior to radiotherapy alone for patients with advanced NPC (26). Two clinical studies showed that synchronous chemotherapy combined with adjuvant chemotherapy significantly improves the survival of patients with locally advanced NPC (27, 28). Two meta-analyses showed that adding chemotherapy to radiotherapy significantly improves the survival rate of patients with locally advanced NPC (29, 30). Sun Y et al. showed that for patients with locally advanced NPC, adding induction chemotherapy based on chemoradiotherapy leads to higher tumor control rates and survival rates (31). Zhang, Y et al. found that the combination of gemcitabine and cisplatin as an induction chemotherapy regimen can significantly improve OS in patients with locally advanced NPC (32, 33). Therefore, Chen, Y. P et al. developed evidence-based recommendations for chemotherapy combined with radiotherapy for the treatment of stage II-IVa NPC (34). In addition, results from a retrospective study of patients with stage III-IVa NPC showed that although there was no difference in OS, progression-free survival (PFS), and local progression-free survival (LRFS) between neoadjuvant chemotherapy (NCT) + CCRT and NCT + IMRT in the low-risk group of EBV DNA cutoff values, patients receiving NCT + CCRT treatment have a better distant metastasis-free survival rate (DMFS) than those receiving NCT + IMRT treatment (35). In pediatric patients with advanced NPC, the use of NCT + CCRT regimen tends to improve long-term DMFS and has acceptable toxicity (36). A recent meta-analysis showed that the addition of induction or adjuvant chemotherapy to CCRT enhances overall survival in NPC compared to chemotherapy alone (37). However, the inclusion of chemotherapy has led to an increase in acute toxicities (38). Results from a recent randomized clinical trial indicated that patients with locally advanced NPC treated with induction chemotherapy followed by radiotherapy alone achieved a 3-year progression-free survival rate that was non-inferior to that of CCRT (39). Another study found that patients with NPC at stage II and T3N0 who exhibit adverse characteristics (defined as: lymph node size ≥ 3cm, radiological evidence of extranodal extension, or Epstein-Barr virus DNA titer ≥ 4000 copies/mL), IMRT combined with chemotherapy did not improve survival rates but led to increased acute toxicities (40). Consequently, the treatment paradigm for NPC has evolved from exclusive radiotherapy to CCRT, followed by the addition of induction and adjuvant chemotherapy. Currently, researchers are focusing on the adverse effects of chemotherapy, aiming to assess through rigorous clinical trials which patients with NPC may benefit from chemotherapy.

Although radiation therapy and chemotherapy have achieved strong therapeutic effects, there are still patients who progress to treatment failure, including recurrence and metastasis. Recent clinical trials showed that nimotuzumab (a humanized monoclonal antibody targeting EGFR) has certain advantages in treating patients with locally advanced NPC, or in combination with chemotherapy, radiotherapy, and CCRT for patients with recurrent or metastatic NPC (41–43). In addition, immunotherapy showed good efficacy in treating patients with recurrent/metastatic NPC (44–46). Thus, targeted therapy and immunotherapy provide another strategy for patients with NPC.

#### 4.3.2 Staging and diagnosis

The prognosis of NPC is closely related to its staging. The 5-year OS of patients with early-stage (I-II) NPC is as high as 94%, while the 5-year survival rate of patients with advanced-stage (III-IV) has sharply decreased, below 80% (47). Lee AW et al. believed that improving baseline assessment level is crucial for distant metastasis of NPC, and magnetic response imaging (MRI) achieves significantly better results than those staged by computed tomography (48). Another prospective clinical study showed that the staging of NPC after neoadjuvant therapy based on MRI determines the OS (49). Recent research indicates that PET/CT offers more precise imaging for patients with locoregional recurrent NPC (RIII-IVA) (50). Additionally, the recursive partitioning analysis (RPA) staging model, which incorporates cell-free Epstein-Barr virus (cfEBV) DNA, lactate dehydrogenase (LDH), and C-reactive protein-to-albumin ratio (CAR) along with TNM staging, surpasses the current TNM system in prognostic prediction and clinical decision-making (51). In a separate proposal, researchers have proposed a more refined classification of T, N, and M: reclassifying T3 NPC cases with early skull base invasion as T2; elevating N1-N2 cases with level 3 extranodal extension (ENE) to N3; combining T2N0 and T1N0 into a single level IA; and subclassifying newly diagnosed metastatic NPC (M1) into M1a (1-3 non-liver-involved metastatic lesions) and M1b (>3 metastatic lesions or liver involvement) (52). Therefore, developing a more suitable staging approach for NPC greatly benefits patient treatment.

Unfortunately, despite significant progress in the treatment of NPC, due to the lack of specificity in the early symptoms (headache, nasal congestion, nosebleeds, etc.), over 60% of patients are diagnosed with advanced stage at the initial treatment, resulting in extremely poor prognosis (53). Therefore, identifying biomarkers with early diagnostic significance is currently a research hotspot. EBV is a key factor causing NPC, so it has always been the subject of research. Lo, Y. M et al. observed a rapid decrease in plasma EBV DNA concentration in patients with NPC after radiation therapy (54). Pre-treatment EBV DNA level is an important risk factor for distant metastasis (55). In addition, studies showed that plasma EBV DNA levels can help screen for early asymptomatic NPC and predict treatment outcomes (56, 57). At present, plasma EBV-DNA has been used for population screening, prognosis, and prediction of treatment response to adapt to treatment and disease monitoring (3). A meta-analysis compared the diagnostic efficacy of 14 diagnostic biomarkers for NPC. The results showed that VCA-IgG had the highest combination sensitivity, EA-IgG had the highest combination specificity, and ZTA-IgG had the highest combination AUC value (58). However, there is heterogeneity in the results of this study, and further exploration is needed in the future.

#### 4.3.3 Micro mechanism

Epithelial-mesenchymal transition (EMT) refers to the biological process in which epithelial cells lose polarity and intercellular adhesion through specific procedures, transforming into cells with mesenchymal phenotype. It is one of the key mechanisms for NPC cells to resist anti-tumor measures, leading to metastasis and invasion (59). Multiple studies have confirmed that TGF-β (60), NF-κB (61), Wnt (62), Akt (63), Notch (64), and other signaling pathways are involved in EMT in NPC, while EBV infection (65), abnormal gene expression (66), hypoxia (67), and abnormal expression of non-coding RNA (68) are involved in regulating these signaling pathways. Stanniocalcin-2 promotes cell EMT by activating the ITGB2/FAK/SOX6 signaling pathway in NPC (69). MiR-296-5p inhibits EMT-related NPC metastasis by targeting TGF-β (70). Circular RNA CRIM1 promotes EMT and chemotherapy resistance in NPC by upregulating FOXQ1 (71). In addition, metabolic reprogramming promotes NPC metastasis by promoting TGFβ1-induced EMT (72). Moreover, EMT is reversible: tumor cells undergo EMT before metastasis, and tumor cells colonize in distant sites and then undergo a mesenchymal-epithelial transition (MET), which then exhibits more malignant behaviors (73). Recent research results showed that EBV-encoded LMP1 and LMP2A coordinate to produce different EMT states in NPC cells, leading to tumor initiation, angiogenesis, and metastasis (74). Therefore, this precise regulation of EMT is crucial for the occurrence and development of NPC.

Cancer stem cells (CSCs) are stem-like cells located at the top of the NPC niche. Although the number of CSCs is rare, their characteristics of tumor initiation, self-renewal, differentiation potential, plasticity, formation of microenvironment, promotion of metastasis, treatment resistance, and immune escape endow them with strong carcinogenic potential (75). Based on these characteristics, CSCs can maintain tumor heterogeneity and protect them from the killing effects of current therapeutic methods (76). Therefore, although patients with NPC actively undergo anti-tumor therapy, most of them still suffer from the painful experience of recurrence and metastasis caused by nasopharyngeal carcinoma stem cells (NPCSCs) (77). Early studies have found that CD44^+^ and aldehyde dehydrogenase 1 (ALDH1) are associated with the recurrence and metastasis of NPC, and they have been identified as specific markers of NPCSCs (78, 79). The embryonic stem cell markers SOX2, OCT4, and Nanog have also been shown to be associated with the EMT in NPC (80). Recent studies have found that the protein C receptor (PROCR), which is associated with poor prognosis in patients with NPC, has the potential to maintain the stemness of tumor cells by regulating lipid metabolism and mitochondrial fission (81). CD166 induces the formation of CSCs by activating the EGFR/ERK1/2 signaling pathway in NPC cells (82). It is noteworthy that EMT and CSCs are interconnected: cancer cells acquire CSCs stemness through EMT processes, while CSCs can also regulate EMT (83, 84). For example, EBV latent membrane protein promotes EMT in NPC cells by activating mTORC1 and mTORC2 pathways, which endows NPC cells with stemness characteristics and promotes cancer cells migration and invasion (85, 86). SLC27A6 promotes EMT and maintains NPCSCs stemness by upregulating NPC cell lipid uptake (87). However, there is evidence that complete EMT is not the best state for maintaining the stemness of CSCs (88). Therefore, in-depth exploration of EMT and CSCs may bring hope to researchers in overcoming the problems of proliferation, metastasis, and drug resistance in NPC.

MicroRNA (miRNA) is a non-coding single-stranded RNA molecule with a length of approximately 22 nucleotides, playing a crucial role in various biological processes (89). MiRNA is a potential biomarker for predicting prognosis and diagnosing radiotherapy resistance in NPC (90, 91). Transactivated MIR106A-5p exerts the effect of macroautophagy/autophagy inhibitors by targeting BTG3 (BTG anti-proliferative factor 3) and activating autophagy to regulate MAPK signaling, thereby promoting the malignant phenotype of NPC (92). EBV-miRNA-BART2-5p directly targets RNase III endonuclease DICER1, inhibiting its ability to cleave double-stranded stem ring RNA into short double-stranded RNA, leading to changes in the expression of a series of key EMT molecules (93). EBV-miR-BART11 and EBV-miR-BART17-3p promote tumor immune escape by inhibiting FOXP1 and PBRM1 and enhancing PD-L1 transcription (61). The expression of let-7i-5p is significantly increased in NPC tissues, and it promotes malignant phenotype by targeting ATG10 and ATG16L1 to inhibit autophagy (62). Lin C et al. found that EBV-encoded miRNA BART8-3p is upregulated in NPC and its expression level in plasma is associated with patient diagnosis and prognosis. Therefore, miRNA is a promising biomarker (94).

Long non-coding RNA (lncRNA) is typically defined as a non-protein coding RNA molecule with a length exceeding 200 nucleotides. LncRNA promotes the occurrence and development of cancer by activating proliferation, migration, and invasion signals, causing cellular energy metabolism disorders, acquiring cancer stem cells characteristics, and regulating microenvironments (95). LncRNA FAM225A promotes NPC tumor occurrence and metastasis by acting as ceRNA on sponge miR-590-3p/miR-1275 and upregulating ITGB3 (96). LINC00173 promotes NPC cell proliferation, migration, and invasion by directly binding and interacting with RAB1B, promoting the secretion of PA2G4 and SDF4 through the exocytosis pathway (97). Wilms tuber 1-associated protein (WTAP) maintains the stability of lncRNA DIAPH1-AS1 in an m6A-dependent manner, promotes the formation of MTDH-LASP1 complex, protects LASP1 from ubiquitin degradation, and promotes the growth and metastasis of NPC (98). It is worth noting that the positive diagnostic rate of tumor-associated platelets (TEP) lncRNA-ROR for NPC is similar to EBV DNA (58.3%), and the positive rate of TEP lncRNA-ROR combined with EBV DNA for NPC can reach 74% (99). Therefore, LncRNA may provide direction for the diagnosis and treatment of NPC.

Exosomes are small (30-100nm) extracellular vesicles originating in the plasma membrane. Studies showed that exosomes mediate tumor immune microenvironment (100), enhance angiogenesis (101), promote metastasis (102), and promote chemoradiotherapy resistance to NPC (103). Wu A et al. showed that exosome LBH inhibits EMT and angiogenesis of NPC by regulating VEGFA expression (104). Jiang J et al. showed that exosomal miR-197-3p (EXO-miR-197-3p) reduces the proliferation, migration, and radiation resistance of NPC cells by regulating AKT/mTOR phosphorylation activation and HSPA5 mediated autophagy (105). Exosomes derived from γδ- T cells (γδ- T Exos) combined with radiotherapy can overcome the radiotherapy resistance of NPC stem cells (106). It is worth considering that extracellular vesicles can carry miRNA, DNA, metabolites, and small molecule drugs, and can accurately transport the carried “goods” from parent cells to recipient cells (107). This provides ideas for the precise treatment of NPC. For example, miRNAs with anti-cancer and improved drug resistance can be loaded into exosomes, which are then taken up and expressed by NPC cells, ultimately enhancing the therapeutic effect (108).

#### 4.3.4 Macro characteristics

Although a large number of articles related to NPC have been published, due to the complexity of this tumor, researchers still can not get a glimpse of its full picture. We have paid attention to some recently published literatures and found that researchers are increasingly concerned about the integrity of cancer: cancer is not only a local disease, but a systemic disease that interacts with various physiological systems and the external environment of the body (109). It is worth noting that the ecological theory of NPC is a new theory with historical significance. From a macro perspective, this theory defines NPC as a multi-dimensional spatio-temporal “ecological and evolutionary unity” disease: an evolutionary adaptive pathological ecosystem composed of four interdependent parts: primary ecosystem, circular ecosystem, transfer ecosystem, and multi-directional ecosystem (110). The core connotation of this system is evolution and adaptability. Specifically, NPC cells produce heterogeneity, metastasis, drug resistance, etc. through intraspecific competition, species evolution, and interspecific interaction, etc. For example, in terms of metabolism, primary tumor cells and circulating tumor cells show “Warburg effect” phenotype and “anti-Warburg effect” phenotype respectively, to adapt to different peripheral environments and achieve the purpose of proliferation and metastasis (111). In terms of immunity, macrophages and neutrophils can be induced into M2 and N2 phenotypes to help tumor cells achieve immune escape (112, 113). In terms of drug resistance, tumor repopulating cells (TRCs) with stem cell-like cancer cell characteristics resist chemotherapy and radiotherapy by reducing the sensitivity to iron death (114). Based on such an ecological theory, researchers can consider using ecological therapies to combat NPC (by altering the surrounding environment to eliminate specific targets), such as destroying tumor habitats through hyperthermia (68) and reducing drug resistance in NPC cells through adaptive therapies (115). Therefore, it is helpful for researchers to develop more effective anti-cancer strategies to examine NPC in a new system.

### 4.4 Future research trends

This study provides insights into possible future trends and potential impacts of NPC. In the future, The clinical treatment of NPC will change from the overall strategy of the whole population to the specific strategy of the specific population. Developing a more accurate NPC evaluation system will help clinicians complete more accurate staging and make better clinical decisions. With the progress of medicine, the molecular mechanism of NPC has gradually become the focus of research. The study of EMT and CSCs is very important for further understanding the mechanism of recurrence, metastasis, and drug resistance of NPC. Identification of special biomarkers is of great significance for the diagnosis and treatment of NPC. In addition, the global concept of NPC has changed dramatically. Integrating the micro molecular mechanisms of NPC and understanding NPC from a macro perspective will help researchers develop more advanced treatment options.

### 4.5 Advantages and limitations

The significant advantage of our research lies in the extensive analysis of global publications on NPC from the perspective of scientific literature. This study also has some limitations. The first is the selection of a database. Although we used the WoSCC database, one of the most extensive and comprehensive global databases, as the literature source database, it may still lead to omissions in literature retrieval. The second is the retrieval strategy. This study only selected the research published in English and ignored the literature in other languages. This can also lead to selection bias. The third is the retrieval time range. Although we have retrieved some high-quality articles published recently as supplements beyond the statistical time, there are still omissions.

## 5 Conclusion

In summary, we conducted a bibliometric analysis using Citespace, VOSviewer, and R version 4.2.2 to outline the current research situation and development trend of nasopharyngeal carcinoma. The article demonstrates the characteristics of the publication, identifies the most influential countries, institutions, authors, journals, articles, and references, and displays keywords and references with the strongest citation bursts. In addition, we also discussed the research hotspots and trends of NPC. At present, optimizing clinical treatment strategies, exploring the molecular mechanisms, and improving diagnostic and staging methods are current research hotspots. The future research trends will be to find advantageous populations for precise treatment, develop more accurate NPC evaluation systems, clarify the progression mechanism of NPC, search for more favorable diagnostic and therapeutic biomarkers, and interpret NPC from a holistic perspective.

## Data availability statement

The original contributions presented in the study are included in the article/**Supplementary Material**. Further inquiries can be directed to the corresponding author.

## Author contributions

GA: Writing – original draft. JL: Writing – original draft. TL: Writing – original draft. LH: Writing – review & editing. YH: Writing – review & editing.
