# Supplementary material for: Global trends in research of nasopharyngeal carcinoma: a bibliometric and visualization analysis
Source: Front Oncol. 2024 Jul 2;14:1392245. doi: 10.3389/fonc.2024.1392245 (PMC11249725; doi:10.3389/fonc.2024.1392245)
Supplement: Supplementary file 1 [file Table_1.docx]

**Supplementary materials**

**Supplementary Table 1.** The most cited articles regarding NPC research each year from 2000 to 2023.

| **Year** | **Title** | **Authors** | **Journal** | **Citations** | **DOI** |
| --- | --- | --- | --- | --- | --- |
| 2000 | Kinetics of plasma Epstein-Barr virus DNA during radiation therapy for nasopharyngeal carcinoma | Lo, Y. M, et al. | Cancer Research | 248 | \ |
| 2001 | Serologic markers of Epstein-Barr virus infection and nasopharyngeal carcinoma in Taiwanese men | Chien YC, et al. | The New England Journal of Medicine | 234 | 10.1056/NEJMoa011610 |
| 2002 | Epidemiology of nasopharyngeal carcinoma | Yu MC, et al. | Seminars in Cancer Biology | 543 | 10.1016/S1044579X02000858 |
| 2003 | Phase III study of concurrent chemoradiotherapy versus radiotherapy alone for advanced nasopharyngeal carcinoma: positive effect on overall and progression-free survival | Lin, J. C, et al. | Journal of Clinical Oncology | 491 | 10.1200/JCO.2003.06.158 |
| 2004 | Quantification of plasma Epstein-Barr virus DNA in patients with advanced nasopharyngeal carcinoma | Lin, J. C, et al. | The New England journal of medicine | 445 | 10.1056/NEJMoa032260 |
| 2005 | Treatment results for nasopharyngeal carcinoma in the modern era: the Hong Kong experience | Lee, A. W, et al. | International Journal of Radiation Oncology Biology Physics | 383 | 10.1016/j.ijrobp.2004.07.702 |
| 2006 | Chemotherapy in locally advanced nasopharyngeal carcinoma: an individual patient data meta-analysis of eight randomized trials and 1753 patients | Baujat, B, et al. | International Journal of Radiation Oncology Biology Physics | 381 | 10.1016/j.ijrobp.2005.06.037 |
| 2007 | Prospective randomized study of intensity-modulated radiotherapy on salivary gland function in early-stage nasopharyngeal carcinoma patients | Kam, M. K, et al. | Journal of Clinical Oncology | 297 | 10.1200/JCO.2007.11.5501 |
| 2008 | Preliminary results of a prospective randomized trial comparing concurrent chemoradiotherapy plus adjuvant chemotherapy with radiotherapy alone in patients with locoregionally advanced nasopharyngeal carcinoma in endemic regions of china | Chen, Y, et al. | International Journal of Radiation Oncology Biology Physics | 131 | 10.1016/j.ijrobp.2007.12.028 |
| 2009 | Intensity-modulated radiation therapy with or without chemotherapy for nasopharyngeal carcinoma: radiation therapy oncology group phase II trial 0225 | Lee, N, et al. | Journal of Clinical Oncology | 399 | 10.1200/JCO.2008.19.9109 |
| 2010 | Randomized trial of radiotherapy plus concurrent-adjuvant chemotherapy vs radiotherapy alone for regionally advanced nasopharyngeal carcinoma | Lee, A. W, et al. | Jnci-journal of The National Cancer Institute | 194 | 10.1093/jnci/djq258 |
| 2011 | How does intensity-modulated radiotherapy versus conventional two-dimensional radiotherapy influence the treatment results in nasopharyngeal carcinoma patients? | Lai, S. Z, et al. | International Journal of Radiation Oncology Biology Physics | 474 | 10.1016/j.ijrobp.2010.03.024 |
| 2012 | A prospective, randomized study comparing outcomes and toxicities of intensity-modulated radiotherapy vs. conventional two-dimensional radiotherapy for the treatment of nasopharyngeal carcinoma | Peng, G, et al. | Radiotherapy And Oncology | 326 | 10.1016/j.radonc.2012.08.013 |
| 2013 | Prospective study of tailoring whole-body dual-modality [18F]fluorodeoxyglucose positron emission tomography/computed tomography with plasma Epstein-Barr virus DNA for detecting distant metastasis in endemic nasopharyngeal carcinoma at initial staging | Tang, L. Q, et al. | Journal of Clinical Oncology | 121 | 10.1200/JCO.2012.46.0816 |
| 2014 | Long-term outcomes of intensity-modulated radiotherapy for 868 patients with nasopharyngeal carcinoma: an analysis of survival and treatment toxicities | Sun, X, et al. | Radiotherapy And Oncology | 372 | 10.1016/j.radonc.2013.10.020 |
| 2015 | Chemotherapy and radiotherapy in nasopharyngeal carcinoma: an update of the MAC-NPC meta-analysis | Blanchard, P, et al. | The Lancet Oncology | 379 | 10.1016/S1470-2045(15)70126-9 |
| 2016 | Induction chemotherapy plus concurrent chemoradiotherapy versus concurrent chemoradiotherapy alone in locoregionally advanced nasopharyngeal carcinoma: a phase 3, multicentre, randomised controlled trial | Sun, Y, et al. | The Lancet Oncology | 499 | 10.1016/S1470-2045(16)30410-7 |
| 2017 | Analysis of Plasma Epstein-Barr Virus DNA to Screen for Nasopharyngeal Cancer | Chan, K. C. A, et al. | The New England Journal of Medicine | 167 | 10.1056/NEJMoa1701717 |
| 2018 | Antitumor Activity of Nivolumab in Recurrent and Metastatic Nasopharyngeal Carcinoma: An International, Multicenter Study of the Mayo Clinic Phase 2 Consortium (NCI-9742) | Ma, B. B. Y, et al. | Journal of Clinical Oncology | 151 | 10.1200/JCO.2017.77.0388 |
| 2019 | Gemcitabine and Cisplatin Induction Chemotherapy in Nasopharyngeal Carcinoma | Zhang, Y, et al. | The New England Journal of Medicine | 344 | 10.1056/NEJMoa1905287 |
| 2020 | Efficacy and Safety of Locoregional Radiotherapy With Chemotherapy vs Chemotherapy Alone in De Novo Metastatic Nasopharyngeal Carcinoma: A Multicenter Phase 3 Randomized Clinical Trial | You, R, et al. | JAMA Oncology | 63 | 10.1001/jamaoncol.2020.1808 |
| 2021 | Chemotherapy in Combination With Radiotherapy for Definitive-Intent Treatment of Stage II-IVA Nasopharyngeal Carcinoma: CSCO and ASCO Guideline | Chen, Y. P, et al. | Journal of Clinical Oncology | 119 | 10.1200/JCO.20.03237 |
| 2022 | Final Overall Survival Analysis of Gemcitabine and Cisplatin Induction Chemotherapy in Nasopharyngeal Carcinoma: A Multicenter, Randomized Phase III Trial | Zhang, Y, et al. | Journal of Clinical Oncology | 18 | 10.1200/JCO.22.00327 |
| 2023 | Camrelizumab Plus Apatinib in Patients With Recurrent or Metastatic Nasopharyngeal Carcinoma: An Open-Label, Single-Arm, Phase II Study | Ding, X, et al. | Journal of Clinical Oncology | 6 | 10.1200/JCO.22.01450 |

**Supplementary Table 2.** The top 10 most cited references regarding NPC research from 2000 to 2023.

| **Rank** | **Title** | **Institution** | **Authors** | **Journal** | **Year** | **Citations** | **DOI** |
| --- | --- | --- | --- | --- | --- | --- | --- |
| 1 | Nasopharyngeal carcinoma | Sun Yat-sen University Cancer Center | Chen YP, et al. | The Lancet | 2019 | 1092 | 10.1016/s0140-6736(19)30956-0 |
| 2 | Chemoradiotherapy versus radiotherapy in patients with advanced nasopharyngeal cancer: phase III randomized Intergroup study 0099 | Providence Cancer Center | Al-Sarraf M, et al. | Journal of Clinical Oncology | 1998 | 1019 | 10.1200/jco.1998.16.4.1310 |
| 3 | Nasopharyngeal carcinoma | University of Hong Kong Medical Centre | Wei WI, et al. | The Lancet | 2005 | 851 | 10.1016/s0140-6736(05)66698-6 |
| 4 | Nasopharyngeal carcinoma | National Cancer Centre Singapore | Chua MLK, et al. | The Lancet | 2016 | 755 | 10.1016/s0140-6736(15)00055-0 |
| 5 | The enigmatic epidemiology of nasopharyngeal carcinoma | Northern California Cancer Center | Chang ET, et al. | Cancer Epidemiology Biomarkers & Prevention | 2006 | 732 | 10.1158/1055-9965.epi-06-0353 |
| 6 | Global cancer statistics 2018: GLOBOCAN estimates of incidence and mortality worldwide for 36 cancers in 185 countries | International Agency for Research on Cance | Bray F, et al. | Ca-a Cancer Journal For Clinicians | 2011 | 666 | 10.3322/caac.21492 |
| 7 | Epidemiology of nasopharyngeal carcinoma | University of Southern California/Norris Comprehensive Cancer Center | Yu MC, et al. | Seminars in Cancer Biology | 2002 | 543 | 10.1016/S1044579X02000858 |
| 8 | Intensity-modulated radiotherapy in the treatment of nasopharyngeal carcinoma: an update of the UCSF experience | University of California-San Francisco | Lee N, et al. | International Journal of Radiation Oncology Biology Physics | 2002 | 536 | 10.1016/s0360-3016(02)02724-4 |
| 9 | Induction chemotherapy plus concurrent chemoradiotherapy versus concurrent chemoradiotherapy alone in locoregionally advanced nasopharyngeal carcinoma: a phase 3, multicentre, randomised controlled trial | Sun Yat-sen University Cancer Centre | Sun Y, et al. | The Lancet Oncology | 2016 | 499 | 10.1016/S1470-2045(16)30410-7 |
| 10 | Phase III study of concurrent chemoradiotherapy versus radiotherapy alone for advanced nasopharyngeal carcinoma: positive effect on overall and progression-free survival | Taichung Veterans General Hospital | Lin JC, et al. | Journal of Clinical Oncology | 2003 | 491 | 10.1200/JCO.2003.06.158 |
