# Supplementary figures and images for: Global trends in research of nasopharyngeal carcinoma: a bibliometric and visualization analysis
Source: Front Oncol. 2024 Jul 2;14:1392245. doi: 10.3389/fonc.2024.1392245 (PMC11249725; doi:10.3389/fonc.2024.1392245)

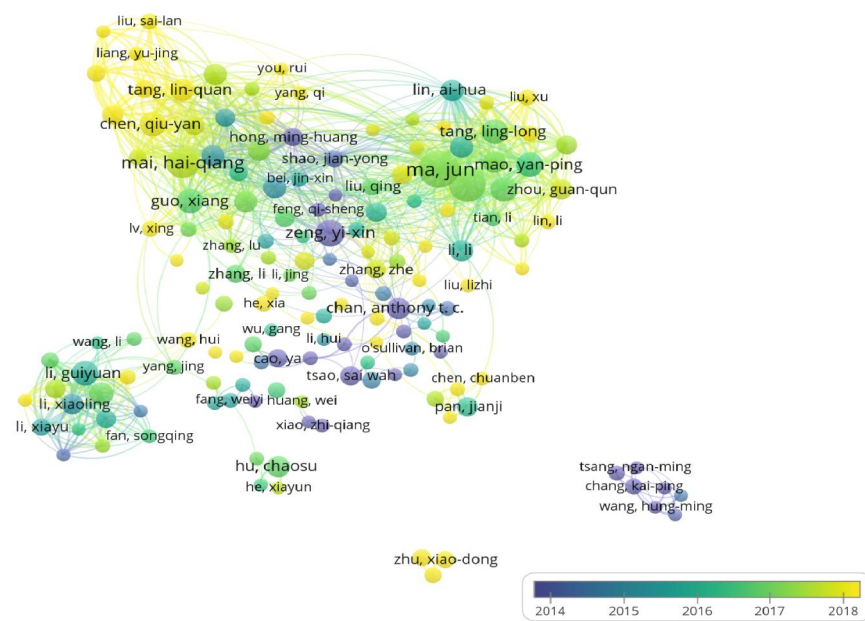

**Supplementary Figure 1.** The coauthorship overlay map of authors.

Supplement: Supplementary file 2 [file DataSheet_1.pdf]
